# The Role of Diet, Alcohol, BMI, and Physical Activity in Cancer Mortality: Summary Findings of the EPIC Study

**DOI:** 10.3390/nu13124293

**Published:** 2021-11-28

**Authors:** Esther Molina-Montes, Esther Ubago-Guisado, Dafina Petrova, Pilar Amiano, María-Dolores Chirlaque, Antonio Agudo, María-José Sánchez

**Affiliations:** 1 Department of Nutrition and Food Science, Faculty of Pharmacy, University of Granada, 18071 Granada, Spain; memolina@ugr.es; 2 Cancer Epidemiology Group, Instituto de Investigación Biosanitaria ibs.GRANADA, 18012 Granada, Spain; dafina.petrova.easp@juntadeandalucia.es (D.P.); mariajose.sanchez.easp@juntadeandalucia.es (M.-J.S.); 3 Epidemiology and Control of Chronic Diseases, CIBER of Epidemiology and Public Health (CIBERESP), 28029 Madrid, Spain; epicss-san@euskadi.eus (P.A.); mdolores.chirlaque@carm.es (M.-D.C.); 4 Institute of Nutrition and Food Technology (INYTA) ‘José Mataix’, Biomedical Research Centre, University of Granada, Avenida del Conocimiento s/n, 18071 Granada, Spain; 5 Escuela Andaluza de Salud Pública, 18011 Granada, Spain; 6 Department of Experimental Psychology, Mind, Brain and Behavior Research Center (CIMCYC), University of Granada, 18071 Granada, Spain; 7 Public Health Division of Gipuzkoa, BioDonostia Research Institute, 20014 Donostia-San Sebastian, Spain; 8 Department of Epidemiology, Regional Health Council, IMIB-Arrixaca, Murcia University, 30003 Murcia, Spain; 9 Unit of Nutrition and Cancer, Catalan Institute of Oncology—ICO, 08908 L’Hospitalet de Llobregat, Spain; a.agudo@iconcologia.net; 10 Nutrition and Cancer Group, Epidemiology, Public Health, Cancer Prevention and Palliative Care Program, Bellvitge Biomedical Research Institute—IDIBELL, 08908 L’Hospitalet de Llobregat, Spain; 11 Department of Preventive Medicine and Public Health, University of Granada, 18071 Granada, Spain

**Keywords:** diet, nutrition, obesity, physical activity, cancer, mortality, prevention

## Abstract

Evidence on the impact of diet, alcohol, body-mass index (BMI), and physical activity on mortality due to cancer and other cancer-related outcomes is still scarce. Herein, we reviewed the contribution of the European Prospective Investigation into Cancer and Nutrition (EPIC) study to the current state of the art on the role of these factors in cancer mortality. We identified 45 studies using a rapid systematic review methodology. Dietary factors associated with reduced cancer mortality included raw vegetable intake; dietary fiber intake; the Mediterranean diet; other dietary scores; other diet patterns including low meat eaters, vegetarians/vegans, or fish eaters; dietary intake (or biomarkers) of some vitamins (e.g., vitamin D, vitamin K2, or Vitamin C); and intake of lignans. Physical activity and following healthy lifestyle recommendations also reduced cancer mortality risk. In contrast, dietary factors associated with higher cancer mortality risk included poor diet quality, consumption of alcohol and soft drinks including juice, and, to a lesser extent, intake of some fatty acids. Excess weight and obesity also increased the risk of cancer mortality. The EPIC study holds valuable information on diet and lifestyle factors and offers a unique opportunity to identify key diet-related factors for cancer mortality prevention.

## 1. Introduction

Cancer is a leading cause of death globally, accounting for over 18.1 million new cancer patients (2.7 million in Europe) and nearly 10 million cancer deaths (1.3 million in Europe) in 2020 [1,2]. Thanks to the improvements in early diagnosis and treatment, survival rates of people with cancer have improved substantially in the last decades and are expected to keep increasing over the coming years [3]. As a result, the population of cancer survivors is growing. They are, however, at increased risk of recurrent tumors and dying from cancer [3]. Overall, cancer is one of the most important public health problems worldwide that also has an important societal and economic impact [1,4].

The World Cancer Research Fund (WCRF) Third Expert Report [5], based on evidence from multiple epidemiologic studies, concluded that diet, nutrition, alcohol intake, excessive body weight, and low physical activity are modifiable risk factors for developing several cancers. To fully understand the impact of these factors on cancer, it is essential to examine their effects on different epidemiological indicators including not only incidence but also mortality. Moreover, it is possible that these risk factors are also linked to cancer-related mortality, something that would reinforce cancer prevention recommendations. The evidence in this respect is, however, limited.

With regard to cancer incidence, a recent umbrella review concluded that there was sufficient evidence to conclude that several foods or nutrients were associated with cancer risk [6]. For instance, strong evidence was found for whole grains in relation to colorectal cancer risk. Other plant-based foods (vegetables, legumes, nuts and seeds, cereals, and vegetable oils), which are rich in fiber and other bioactive compounds, are also likely to play an inhibitory role in several carcinogenic mechanisms [7].

Drinking alcohol has also been shown to be associated with an increased risk of cancer in general [8], causing an estimated 376,200 cancer deaths worldwide in 2016 (about 80,000 in Europe), representing 4.2% of all cancer deaths [9]. Alcoholic beverages contain numerous carcinogenic compounds, but ethanol is thought to be the one that explains most of the increase in cancer risk [10]. 

The effect of increased body mass index (BMI) on cancer risk has been studied thoroughly; however, this factor has rarely been examined in relation to cancer mortality [11,12,13,14]. The potential harmful effect of high BMI on cancer risk and mortality appears to be due to a genotoxic stress produced by a state of chronic inflammation in adipose tissue, contributing to carcinogenesis and cancer progression [15]. Obesity and sedentary lifestyle are estimated to cause about 25% of all cancer worldwide [16]. In contrast, an increase in regular physical activity is related to a decrease in the development of numerous types of cancer [17]. There are many potential mechanisms that link physical activity to a decreased cancer risk: a lower systemic inflammation, insulin-like growth factor (IGF-I), hyperinsulinemia, pro-inflammatory leptin, sex hormones, other obesity-related cytokines, and an increment in anti-inflammatory adiponectin levels [18]. 

As aforementioned, whereas the impact of lifestyle factors on cancer risk has been examined in multiple studies, their relationship with cancer mortality and other cancer-related outcomes remains poorly understood [5]. One reason is that large and well-powered studies are necessary to reliably investigate their role in cancer mortality [19]. It is possible that lifestyle factors are associated with cancer risk and cancer mortality in very similar ways. However, it is also possible that their effect on incidence and mortality from cancer differs due to the specific types of tumors they might be associated with or the effectiveness of cancer treatments, among others [19]. In view of this fact, it seems necessary to study in more detail the impact of dietary factors, body composition, and physical activity on cancer mortality and other cancer outcomes, all being potential targets for prevention interventions. 

The European Prospective Investigation into Cancer and Nutrition (EPIC) is one of the largest cohort studies worldwide. Its aim is to investigate the relationship between diet, lifestyle, and environmental factors with the risk of developing cancer (and other chronic diseases). It is a large multi-center prospective cohort study comprising 519,978 participants (153,457 men and 366,521 women), mostly aged 35–70 years, who were enrolled between 1992 and 1998 [20,21]. The study recruitment was carried out in 23 centers in 10 European countries: Germany, Denmark, Italy, France, Greece, the Netherlands, Spain, Norway, the United Kingdom, and Sweden. The participants were mostly selected from the general population, except for Utrecht and Florence (women attending breast cancer screening), France (women who were health insurance members), Oxford (mostly health-conscious volunteers including a large proportion of vegetarians), and some centers in Spain and Italy where participants were mostly blood donors.

Thus, the study included populations with high heterogeneity in dietary habits and the incidence of several major cancer sites [22]. Thanks to these features, the EPIC study has contributed significantly to the scientific body of knowledge regarding the role of diet and other risk factors in cancer prevention, and many of its findings have served to establish international recommendations on cancer prevention [5]. 

The evidence from EPIC regarding the role of diet in cancer prevention was last summarized more than 10 years ago [23]. Since then, a large number of publications about new diet-related exposures and with longer follow-up have been published. As part of a special issue on “Diet and Nutrition in Cancer Epidemiology”, we set out to summarize the evidence generated by the EPIC study regarding the influence of diverse lifestyle factors on cancer outcomes. Following a previous review focused on diet and cancer incidence [24], this review aimed to summarize the findings derived from the EPIC study regarding the associations between dietary factors and other lifestyle exposures such as alcohol, BMI, and physical activity with cancer mortality.

## 2. Materials and Methods

A rapid review was conducted to synthesize the knowledge and to speed up the process of conducting a traditional systematic review by simplifying or omitting specific methods, in order to produce evidence for stakeholders in a resource-efficient manner [25].

**Figure 1:**
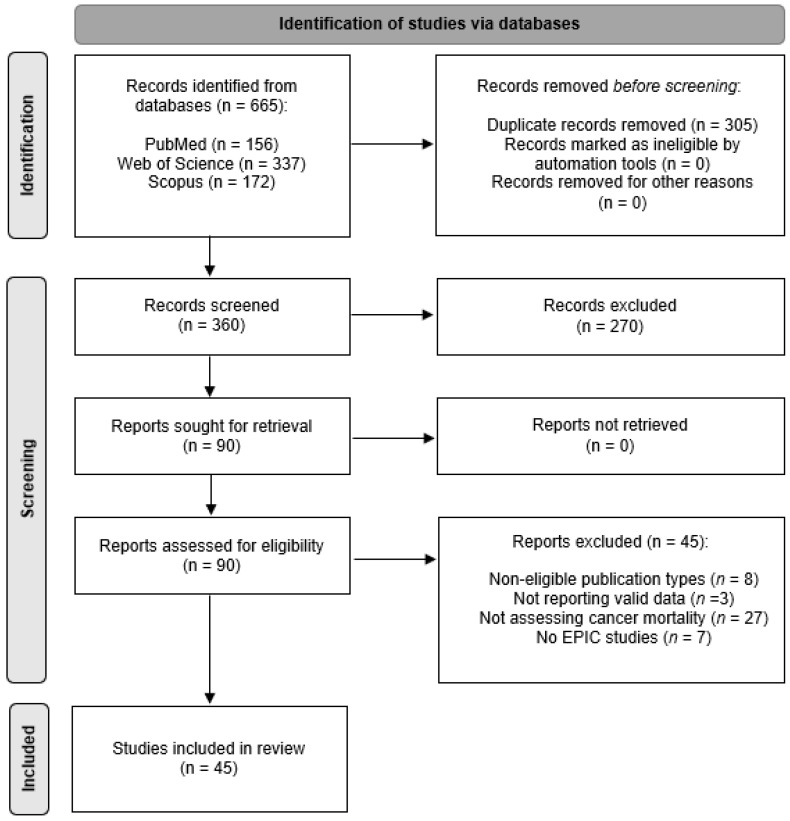
Preferred Reporting Items for Systematic Reviews and Meta-Analyses (PRISMA) flow diagram of study selection, inclusion, and exclusion.

The general methodology of the Preferred Reporting Items of Systematic Reviews and Meta-Analyses (PRISMA) guidelines (Figure 1) was applied [26]. According to PICOS, the following elements were used to frame the study question:

Population: Adults participating in the EPIC study and/or cancer patients.
Interventions: exposure to diet and diet-related factors.
Comparisons: differences in survival and other outcomes between cancer and non-cancer subjects by the exposure factors.
Outcomes: Cancer mortality, Cancer cause-specific mortality, and other cancer-related outcomes.
Study design: longitudinal studies (cohort, nested case-control, or case-cohort studies).

### 2.1. Search Strategy

A systematic search in MEDLINE (via PubMed), Scopus, and Web of Science of articles based on data from the EPIC study was undertaken between 1 and 30 September 2021. The search strategy included the following terms: (“European prospective investigation into cancer” OR “European prospective investigation into cancer and nutrition” OR “EPIC study”) AND (“cancer” OR “tumor” OR “tumour” OR “myelo*” OR “leukaemia” OR “leukemia” OR “neoplasm*” OR “lympho*” OR “carcinoma” OR “sarcoma”) AND (“diet” OR “intake” OR “nutrients” OR “physical activity” OR “exercise” OR “BMI” OR “alcohol”) AND (“mortality” OR “survival”). The complete search strategies used for each database are available in Table S1. 

### 2.2. Study Elegibility Criteria

The review considered original studies conducted on the EPIC cohort. All included studies were to be prospective cohort studies, nested case-control studies, or case-cohort studies. Narrative reviews were not eligible, but they were considered to retrieve potentially relevant studies by manual search.

*Study outcomes*: We included studies on overall and/or cancer-specific mortality and studies reporting results of other cancer-related outcomes such as recurrence and progression of the disease. Studies examining associations with cancer mortality with follow-up starting both pre-diagnosis (i.e., on the date of recruitment into EPIC) and post-diagnosis (i.e., on the date of cancer diagnosis) were included.

*Exposures*: We included studies examining the influence of dietary factors (food, food groups, nutrients, biomarkers, and dietary patterns), alcohol intake, body composition, and physical activity.

*Exclusion criteria*: Studies that did not report any risk estimate (odds ratio, OR; relative risk, RR; or hazard ratio, HR; and the corresponding 95% confidence interval, CI) regarding the association between the factors and outcomes of interest were excluded. 

### 2.3. Data Collection and Analysis

Studies were first screened by title and abstract by two reviewers (E.M.M. and E.U.G.) and the final study selection was performed based on a full text review. Any discrepancies were resolved by consulting a third reviewer (M.J.S.).

### 2.4. Data Extration and Management

Data extraction was performed by two reviewers (E.M.M. and E.U.G.) using a predefined standardized form to collect information on (1) study characteristics: authors and year(s), study population characteristics with regard to sample size (number of fatal cases and total population size), and follow-up to distinguish between pre- and post-diagnosis cancer mortality association studies; (2) the exposure factor under consideration: dietary factors or other lifestyle exposures (alcohol, body composition, and physical activity); (3) the outcomes: overall cancer mortality, cancer-specific mortality, and other cancer-related outcomes; and (4) the reported results: measures of effect size (OR, HR, and RR, with 95% CI) from multivariate adjusted models, comparing high versus low levels of exposure, or at the continuous scale by considering increasing levels of exposure.

Given that all studies were based on EPIC study samples, we did not collect information on exposure and outcome assessment. This information was common to all studies:

(1) Regarding exposure assessment: Diet information regarding the previous 12 months was collected at the time of recruitment using validated country/center-specific dietary questionnaires [27,28]. According to each center’s protocol, self-administered quantitative or semi-quantitative methods were applied: food-frequency questionnaires (with approximately 260 food items), diet history questionnaires (with more than 600 food items) administered by means of interviews, and semi-quantitative food-frequency questionnaires combined with dietary record [21]. To calibrate the dietary measurement and to correct the errors produced by overestimation or underestimation of food intake, a 24-h recall was performed by a computerized program (EPICSOFT) in a random subsample of 8% of the cohort [29]. Nutrients were analyzed using a standardised Food Composition Table (EPIC Nutrient Database ENDB) [30]. Lifestyle questionnaires were used to obtain information on education, habits, lifestyles, and medical history. Anthropometry (height, weight, waist, and hip circumference) was measured using standard study protocols [22]. For example, weight and height was measured in light clothing without shoes. Information on physical activity was gathered by means of a validated questionnaire using accelerometers [21,22].
(2) Regarding outcome assessment: Information on deaths occurred during follow-up (the date and underlying cause of death) was obtained through linkage to national or regional mortality registries or active follow-up (in France, Germany, and Greece), depending on the study center. The 10th edition of the International Classification of Diseases (ICD-10) [31] was used to define cause-specific mortality. For post-diagnosis association studies, the date of cancer diagnosis was used as the start date of follow-up (i.e., from diagnosis to death), whereas for pre-diagnosis association studies the follow-up was started at the date of recruitment (i.e., from recruitment to death). Of note, no study accounted for exposure assessments at the date of diagnosis; thus, all association studies were based on diet and lifestyle habits long before the diagnosis of cancer. Participants were censored at date of death, last date of contact, or the date at which follow-up data were considered to be complete at each study center.

### 2.5. Quality Assessment

In the previously mentioned EPIC review on cancer incidence [24], the methodological assessment resulted in generally high and highly homogenous methodological quality scores; similarly, in the current review, a methodological quality assessment was performed using the same tool (Joanna Briggs Institute Critical Appraisal Tool for Systematic Reviews) [32]. This tool considers 11 items, each of them with four possible answers: “yes” (criterion met), “no” (criterion not met), “unclear”, and “not applicable” (N/A). A study was considered as “high quality” when the quality score was at least 0.75 (i.e., 75%), whereas studies were considered as “low quality” when the quality score was lower than 0.75. In addition, a score for each criterion was calculated by dividing the number of positively scored by the total number of included studies, to provide an overview of how well the current literature scores on each criterion.

### 2.6. Presentation of Results

The results of all studies were presented in tabular format and summarized narratively according to (1) the type of association examined (protective or risk factors), (2) the type of factor (dietary factors and other lifestyle exposures), and (3) the cancer-related outcome (overall mortality and cause-specific mortality). To summarize the findings, we described their results and risk estimates accompanied by 95% confidence intervals adjusted for all potential confounders, as reported in the studies.

## 3. Results

**Figure 2:**
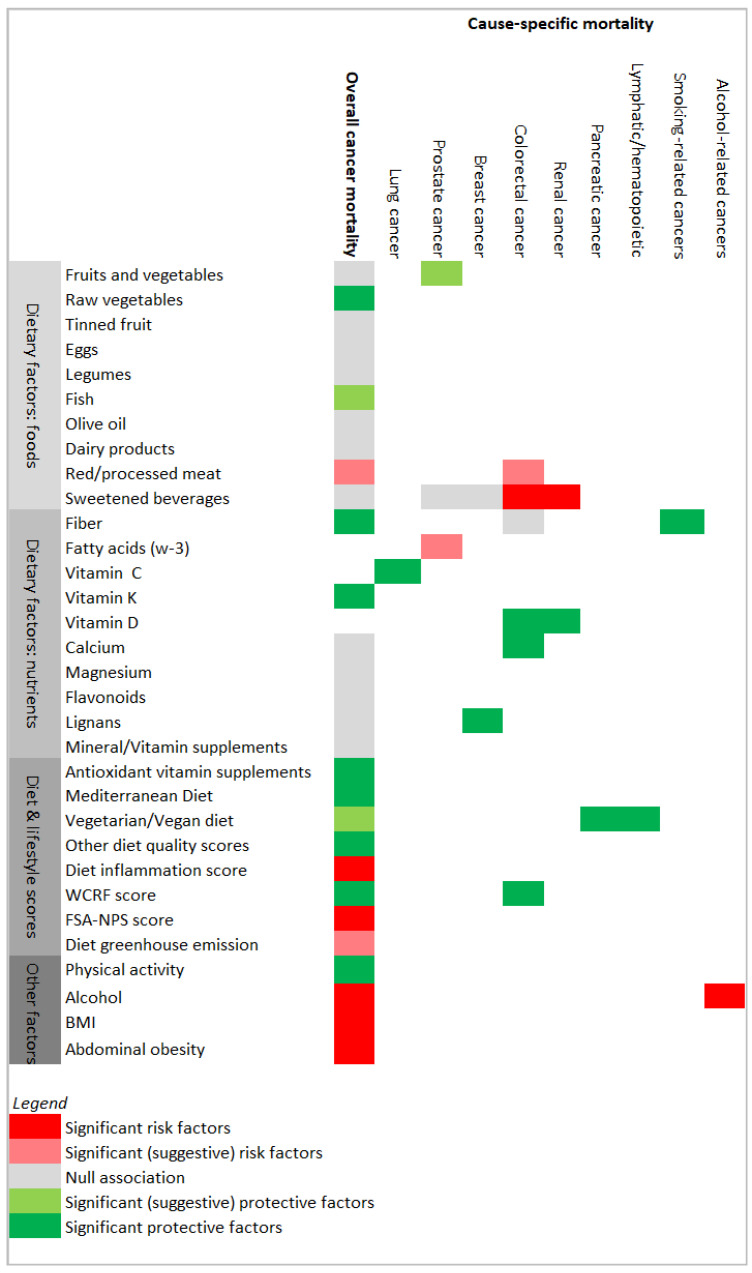
Results’ matrix showing associations between the exposure factors and cancer mortality, overall and by type of cancer. Potential risk factors or protective factors are colored via red or green color scales, respectively. FSA-NPS score (Food Standards Agency nutrient profiling system dietary index score); WCRF score (World Cancer Research Fund score). “Other diet quality scores” included the Diet Quality Index–International, the Healthy Nordic Food Index, the Healthy Eating Index 2010, and the Dietary Approaches to Stop Hypertension score, as described in the Results section.

The results were summarized according to exposure factors and are illustrated in Figure 2.

### 3.1. Study Quality

All studies that entered the review (see Figure 1) were high-quality studies according to the assessment tool for Systematic Reviews from The Joanna Briggs Institute (Table S2) [32]. Table S3 shows the percentage of studies meeting the quality criteria and provides detailed information on the quality score of each study. As observed previously [24], high-quality scores were reached given that the study populations emerged from the same study (the EPIC study), considering the same research protocols and data.

When the studies were analysed by individual domains, 100% of the studies measured the exposure and the outcomes in a valid and reliable manner, identified the potential confounders, and took them into account within the study design or in the data analysis. The participants were free of the outcomes of interest at the start of the study. The follow-up was completed by a large percentage of participants, and the statistical analysis used was appropriate. It should be taken into account that, among the studies that had two groups, 100% of them had similar groups that were recruited from the same population, and 100% of those studies measured the exposures similarly in order to assign people to the exposed or unexposed groups. In no study was it necessary to apply strategies to address incomplete follow-up.

### 3.2. Protective Factors

We identified 27 studies reporting associations between dietary factors (Table 1) and other lifestyle exposures (Table 2) hypothesized to be protective factors against cancer mortality.

*Foods from plant sources:* No study observed significant associations between fruit and vegetable consumption (combined or separately) and overall cancer mortality [33,34] or prostate cancer mortality [35]. Only one significant association was found between raw vegetable intake and overall cancer mortality: HR 0.90 (0.84–0.96) [34]. There was also a non-significant association between intake of legumes and cancer mortality risk [33]. A borderline protective effect was found between intake of dietary fiber and mortality from all cancers combined [36] and smoking-related cancers [37] ( HR 0.82 (0.66–1.02) and HR 0.89 (0.80–0.99), respectively), but not against mortality from colorectal cancer [38].

*Foods from animal sources:* There were no significant associations between consumption of total fish, lean, or fatty fish and overall cancer mortality [39]. Intake of dairy products (intake of milk, yogurt, cheese, butter, calcium from dairy, full-fat milk, or reduced-fat milk) was also not associated with cancer mortality [40].

*Diet patterns:* A higher adherence to the Mediterranean diet had a borderline protective effect (HR 0.79, 0.61–1.01, *p* = 0.056) against mortality from cancers, with greater evidence of being causally related to dietary factors (esophageal, stomach, intestinal, colorectal and other digestive organ cancers, pancreatic, breast, and prostate cancer) but not against mortality from cancer overall [41]. However, the association between the Mediterranean diet and cancer mortality was found to be significant in another study [42] that showed protective effects on overall cancer mortality of various Mediterranean dietary scores, such as the Mediterranean Diet Scale, relative Mediterranean diet score, or Mediterranean Style Dietary Pattern Score. 

Low meat eaters and vegetarians/vegans compared with regular meat eaters experienced a significant reduction of pancreatic cancer mortality (HR 0.55 (0.36–0.86) and HR 0.48 (0.28–0.82), respectively) [43], but not of overall cancer mortality [44]. Similar findings were reported for vegetarians/vegans [43] and fish eaters [44] (compared with regular meat eaters) in relation to mortality from cancers of the lymphatic/hematopoietic tissue [43] and all cancers combined [44]: HR 0.50 (0.32–0.79), and HR 0.83 (0.70–0.97), respectively. Finally, other dietary scores showed protective effects against overall cancer mortality including the Diet Quality Index–International, the Healthy Nordic Food Index, the Healthy Eating Index 2010, and the Dietary Approaches to Stop Hypertension score.

*Physical activity:* Physical activity levels of a minimum of 150 min/week of moderate-intensity physical activity compared to being inactive had a protective effect against overall cancer mortality: HR 0.89 (0.79–0.99) [45]. By specific sport practice, no significant associations were found with cycling up to 1 h/week or cycling for more than 1 h per week [46]. Household physical activity was also a protective factor for overall cancer mortality: HR 0.72 (0.54–0.94) in men and HR 0.52 (0.34–0.79) in women [47]. 

*Healthy lifestyle recommendations:* Adherence to the WCRF recommendations showed a reduced risk of mortality for all cancers [42,48]: HR 0.90 (0.88–0.92), HR 0.80 (0.69–0.93), and rectal cancer [49]: HR 0.70 (0.56–0.89), respectively. In addition, high adherence to the Healthy Lifestyle Index was also associated with lower overall cancer mortality: HR 0.80 (0.78–0.82) [42]. 

*Other dietary exposures:* Some vitamins showed associations with overall or specific cancer mortality. A protective effect between plasma levels of 25(OH)D (vitamin D) and colorectal (HR 0.69, 0.50–0.93) [50] or renal [51] cancer mortality was found. By tumor location, in colorectal cancer, higher 25(OH)D levels were associated with reduced mortality due to rectal cancer: HR 0.48 (0.29–0.80) [50]. Furthermore, participants with high dietary calcium intake (≥928 mg/d) and high pre-diagnosis vitamin D levels (>100 nmol/L) had a lower risk for colorectal cancer mortality compared to those presenting the lowest 25(OH)D levels (<25 nmol/L): HR 0.24 (0.11–0.54) [50]. A diet rich in menaquinones (vitamin K2) significantly reduced the risk of overall cancer mortality: HR 0.72 (0.53–0.98) [52]; however, a later study found no association with either overall cancer or lung cancer mortality [53]. High vitamin C plasma levels were also related to lung cancer mortality [54]: HR 0.54 (0.35–0.81). Another study did not find significant associations between vitamin/mineral supplementation or multivitamin supplementation with overall cancer mortality [55]. However, users of antioxidant vitamin supplements at baseline had a significantly reduced risk of overall cancer mortality: HR 0.52 (0.28–0.97) [55]. Non-users who started taking vitamin/mineral supplements during follow-up had significantly increased risks of overall cancer mortality: HR 1.74 (1.09–2.77) [55]. Finally, intake of lignans was related with a lower risk of breast cancer mortality in postmenopausal women: HR 0.72 (0.53–0.98) [56]. No associations were found between cancer mortality and intake of calcium [57], magnesium [57], olive oil [58], total flavonoid intake, flavonoid subclasses, or lignin intake [59].

**Table 1:** Dietary factors investigated as potential protective factors in relation to cancer mortality in the EPIC study.

Tumour Site and EPIC Subcohort (If Applicable)	No. of Cases (No. of Deaths)	Mean Follow-Up (Years)	Results, Relative Risk [95% Confidence Interval (CI)]	Reference
Cancer overall among diabetics (confirmed at recruitment)	10,449 (1346 total deaths/319 from cancer)	9.0 years since recruitment	No significant association between intake of total vegetables, legumes, and fruits and cancer mortality risk in subjects diagnosed with diabetes at recruitment (per 80 g/d intake): Vegetables, legumes, and fruits: HR 1.08 (0.99–1.17) Vegetables: HR 1.09 (0.87–1.33) Legumes: HR 1.09 (0.96–1.24) Fruits: HR 1.08 (0.98–1.19)	Nöthlings 2008 [33]
Cancer overall EPIC-Heidelberg	24,340 (458 deaths from cancer)	10.7 years since recruitment	Dietary intake of menaquinones (vitamin K2) was significantly associated with cancer mortality (highest vs. lowest intake): HR 0.72 (0.53–0.98), while intake of phylloquinone (vimtain K1) was not associated with cancer mortality.	Nimptsch 2010 [52]
Cancer overall EPIC-Spain	40,622 (1855 total deaths/913 from cancer)	13.4 years since recruitment	No significant association between adherence to the relative Mediterranean diet score and cancer mortality. However, in analyses including only cancers with greater evidence of being causally related to dietary factors (oesophageal, stomach, intestinal, colorectal and other digestive organ cancers, pancreatic, breast, and prostate cancer) (570 cases), the relative Mediterranean diet score was associated with a borderline reduction in risk of death for high versus low score: HR 0.79 (0.61–1.01) *p* = 0.056	Buckland 2011 [41]
Colorectal cancer Lung cancer Prostate cancer Breast cancer EPIC-Heidelberg	24,323 (1101 total deaths/513 from cancer)	11.0 years since recruitment	No association between dietary intake of calcium and magnesium and cancer-related mortality risk (highest vs. lowest intake): HR 0.90 (0.68–1.20) and HR 1.04 (0.79–1.36), respectively.	Li 2011 [57]
Cancer overall EPIC-Spain	40,622 (1915 total deaths/956 from cancer)	13.4 years since recruitment	No significant association was observed between olive oil and cancer mortality (highest vs. lowest intake): HR 0.90 (0.72–1.13).	Buckland 2012 [58]
Cancer overall among diabetics (confirmed at recruitment)	6192 (791 total deaths/163 from cancer)	9.2 years since recruitment	An inverse, though non-significant, association was observed for dietary fiber in analyses of mortality risk due to cancer: HR 0.82 (0.66–1.02).	Burger 2012 [36]
Cancer overall Smoking-related cancers	452,717 (23,582 total deaths)	12.7 years since recruitment	Dietary fiber intake was not related with risk of death from cancer. An inverse association with smoking-related cancers was found (per 10 g/d increase): HR 0.89 (0.80–0.99).	Chuang 2012 [37]
Colorectal cancer	1202 (541 total deaths/444 from cancer)	6.0 years since diagnosis	Higher 25(OH)D levels were associated with a reduction in colorectal-specific mortality. Participants with 25(OH)D levels in the highest quintile had an adjusted HR of 0.69 (0.50–0.93) for colorectal cancer-specific compared with the lowest quintile. By tumor location, higher 25(OH)D was associated with reduced mortality for rectal cancers, comparing the highest versus the lowest levels: HR 0.48 (0.29–0.80) for colorectal cancer-specific mortality. Participants with high dietary calcium intake (≥928 mg/d) and high pre-diagnosis vitamin D levels (>100 nmol/L) showed a HR of 0.24 (0.11–0.54) for colorectal cancer-specific mortality, compared with participants with the lowest 25(OH)D levels (<25 nmol/L). Among participants with low calcium intake, the corresponding HRs were 0.86 (0.41–1.82) for colorectal cancer-specific mortality compared with participants with the lowest 25(OH)D levels.	Fedirko 2012 [50]
Cancer overall EPIC-Heidelberg	23,943 (1101 total deaths/513 from cancer)	11.0 years since recruitment	Neither any vitamin/mineral supplementation nor multivitamin supplementation at baseline was statistically significantly associated with cancer mortality. However, baseline users of antioxidant vitamin supplements had a significantly reduced risk of cancer mortality: HR 0.52 (0.28–0.97). In comparison with never users, baseline non-users who started taking vitamin/mineral supplements during follow-up had significantly increased risks of cancer mortality: HR 1.74 (1.09–2.77).	Li 2012 [55]
Cancer overall	451,151 (25,682 total deaths/10,438 from cancer)	~13.0 years since recruitment	No association between cancer mortality and fruit and vegetable intake (highest vs. lowest intake): HR 0.96 (0.90–1.03) or intake of vegetables or fruits: HR 0.95 (0.89–1.02) and HR 0.98 (0.92–1.05), respectively. A significant association was seen for raw vegetable intake (highest vs. lowest quartile): HR 0.90 (0.84–0.96), but not for cooked vegetables (highest vs. lowest quartile): HR 0.98 (0.91–1.06).	Leenders 2013 [34]
Cancer overall EPIC-Spain	40,622 (1915 total deaths/956 from cancer)	13.6 years since recruitment	No association between total flavonoid intake, flavonoid subclasses, or lignan intake and cancer-related mortality risk: HR for log2 (doubling of intake of total flavonoids) = 0.96 (0.89–1.04).	Zamora-Ros 2013 [59]
Renal cancer	560 (205 deaths from cancer)	3.2 years since diagnosis	Plasma levels of 25(OH)D3 (vitamin D) were nonlinearly associated with risk of death. High concentrations of pre-diagnostic 25(OH)D3 were associated with decreased hazards of death among renal cancer patients (statistical results not reported).	Muller 2014 [51]
Cancer overall	480,535 (32,587 total deaths)	7.0–18.0 years since recruitment	No associations were found for consumption of total fish, lean, or fatty fish and cancer mortality. However, there seemed to be a U-shaped (*p* = 0.046) trend with total fish consumption in the analyses of cancer mortality (highest vs. lowest intake).	Engeset 2015 [39]
Breast cancer	11,782 (1482 total deaths/753 from cancer)	6.0 years since diagnosis	Among postmenopausal women, an intake of lignans was related to a 28% lower risk of dying from breast cancer (highest vs. lowest intake): HR 0.72 (0.53–0.98).	Kyrø 2015 [56]
Cancer overall Colorectal cancer Pancreatic cancer Lung cancer Breast cancer Ovarian cancer Lympathic/hematopoietic cancer EPIC-Oxford	65,429 (2137 deaths from cancer)	~5.0 years since recruitment	There was a significantly reduced cancer mortality risk due to pancreatic cancer for low meat eaters and vegetarians/vegans (compared with regular meat eaters): HR 0.55 (0.36–0.86) and HR 0.48 (0.28–0.82), respectively. Additionally, for cancers of the lymphatic/hematopoietic tissue, for vegetarians/vegans (compared with regular meat eaters): HR 0.50 (0.32–0.79). Cancer-related overall mortality risk was significantly lower in fish eaters than in regular meat eaters: HR 0.82 (0.70–0.97).	Appleby 2016 [43]
Cancer overall	451,256 (15,200 total deaths/7475 from cancer)	12.8 years since recruitment	All dietary scores showed inverse associations with cancer mortality (highest vs. lowest score). Mediterranean Diet Scale: HR 0.93 (0.91–0.95); relative Mediterranean diet score: HR 0.92 (0.90–0.94); Mediterranean Style Dietary Pattern Score: HR 0.94 (0.92–0.96); Diet Quality Index–International: HR 0.91 (0.89–0.93); Healthy Nordic Food Index: HR 0.95 (0.92–0.97); Healthy Eating Index 2010: HR 0.93 (0.90–0.95); Dietary Approaches to Stop Hypertension: HR 0.94 (0.92–0.96).	Lassale 2016 [42]
Colorectal cancer	3789 (1262 total deaths/1008 from cancer)	4.1 years since diagnosis	No association between pre-diagnostic intakes of fibre (defined as quartiles and continuous grams per day) and death due to colorectal cancer: HR 1.00 (0.87–1.15).	Ward 2016 [38]
Prostate cancer	7036 (936 from cancer)	13.9 years since recruitment	No association between intake of vegetables and fruits with death due to prostate cancer (per 100 g intake): HR 1.11 (0.95–1.30) and HR 0.97 (0.91–1.04), respectively.	Perez-Cornago 2017 [35]
Cancer overall Lung cancer EPIC- Netherlands	33,289 (2863 total deaths/1346 from cancer)	16.8 years since recruitment	None of the forms of vitamin K intake were associated with overall cancer mortality (highest vs. lowest intake): HR 1.01 (0.85–1.19) for phylloquinone, HR 0.96 (0.78–1.18) for menaquinones, HR 1.20 (0.93–1.54) for short chain, and HR 1.01 (0.84–1.23) for long chain. No associations were found between vitamin K intake and lung cancer mortality (highest vs. lowest intake): HR 0.99 (0.86–1.23).	Zwakenberg 2017 [53]
Lung cancer EPIC-Norfolk	19,336 (687 total deaths/280 from cancer)	16.5 years since recruitment	Significant risk reduction of lung cancer mortality for plasma vitamin C concentrations (highest vs. lowest quartile): HR 0.54 (0.35–0.81).	Myint 2019 [54]
Cancer overall EPIC-Italy	45,009 (2468 total deaths/1464 from cancer)	14.9 years since recruitment	No association between intake of dairy products and cancer death (highest vs. lowest intake): HR for intake of milk 1.05 (0.89–1.23), HR for intake of yogurt 1.00 (0.83–1.20), HR for intake of cheese 1.08 (0.88–1.32), HR for intake of butter 0.90 (0.79–1.11), HR for intake of calcium from dairy 1.18 (0.99–1.40), HR for intake of full-fat milk 1.08 (0.89–1.32), and HR for intake of reduced-fat milk 1.10 (0.89–1.35).	Pala 2019 [40]
Cancer overall EPIC-Oxford	65,429 (2137 deaths from cancer)	~5 years since recruitment	Relative to regular meat eaters, a reduced risk of cancer-related mortality was observed for fish eaters (HR 0.83, 0.70–0.97), though not for low meat eaters (HR 0.96, 0.87–1.08), vegetarians (HR 0.91, 0.80–1.03), or vegans (HR 1.14, 0.88–1.47).	Segovia-Siapco 2018 [44]

HR: hazard ratio.

**Table 2:** Other lifestyle exposures investigated as potential protective factors for cancer mortality in the EPIC study.

Tumour Site and EPIC Subcohort If Applicable	No. of Cases (No. of Deaths)	Mean Follow-Up (Years)	Results, Relative Risk [95% Confidence Interval (CI)]	Reference
Cancer overall EPIC-Norfolk	22,450 (4398 total deaths/1639 from cancer)	~7.0 years since recruitment	Relative to non-cycling, cycling up to 1 h/week or cycling for more than 1 h/week were not associated with cancer-related mortality risk: HR 0.99 (0.73–1.34) and HR 0.93 (0.81–1.06), respectively.	Sahlqvist 2013 [46]
Cancer overall	378,864 (23,828 total deaths/9388 from cancer)	12.8 years since recruitment	Adherence to the WCRF recommendations was associated with a reduced risk of cancer-related mortality (highest vs. lowest category): HR 0.80 (0.69–0.93). HR per unit increase in the score = 0.91 (0.89–0.93).	Vergnaud 2013 [48]
Colorectal cancer	3293 (1113 total deaths/872 from cancer)	4.2 years since diagnosis	Adherence to WCRF cancer prevention recommendations was associated with lower risk of rectal cancer mortality (highest vs. lowest category): HR 0.70 (0.56–0.89)	Romaguera 2015 [49]
Cancer overall EPIC-Spain	38,379 (1371 total deaths/758 from cancer)	13.6 years since recruitment	Household physical activity was inversely associated with cancer mortality in men and women (highest vs. lowest levels of MET-h/week): HR 0.72 (0.54–0.94) and HR 0.52 (0.34–0.79), respectively.	Huerta 2016 [47]
Cancer overall	451,256 (15,200 total deaths/7475 from cancer)	12.8 years since recruitment	Healthy Lifestyle Index and WCRF/AICR adherence scores showed significant associations with cancer mortality (highest vs. lowest score): HR 0.80 (0.78–0.82) and HR 0.90 (0.88–0.92), respectively.	Lassale 2016 [42]
Cancer-overall EPIC-Norfolk	14,599 (3148 total deaths/1091 from cancer)	15.0 years since recruitment	Higher physical activity levels (a minimum of 150 min/week of moderate-intensity physical activity vs. being inactive) were inversely associated with cancer-related mortality risk (for each 1 kJ/kg/day per year increase in physical activity energy expenditure): HR 0.89 (0.79–0.99).	Mok 2019 [45]

AICR: American Institute for Cancer Research; HR: hazard ratio; WCRF: World Cancer Research Fund.

### 3.3. Risk Factors

We identified 19 studies reporting associations between dietary factors (Table 3) and other lifestyle exposures (Table 4) hypothesized to be risk factors for cancer mortality.

*Foods from plant sources:* Consumption of tinned fruit was not associated with an increased risk of cancer mortality [60]. 

*Foods from animal sources*: The associations between consumption of different meat types (intake of red meat, processed meat, or poultry) and cancer-related mortality risk were non-significant [38,61]. A borderline significant trend was found only for processed meat and colorectal cancer mortality (*p* = 0.053) [38]. No association was found between egg consumption and cancer-related mortality [62].

*Diet patterns:* Higher scores in the Inflammatory Score of the Diet [63] and higher scores in the Food Standards Agency nutrient profiling system dietary index [64], denoting lower nutritional quality diet in both scores, were associated with a higher risk of mortality for all cancers: HR 1.44 (1.22–1.69) and HR 1.08 (1.03–1.13), respectively. 

*Alcoholic and non-alcoholic drinks:* The risk of mortality for all cancers and alcohol-related cancers increased with alcohol intake [65]: HR in men = 1.34 (1.13–1.59) for all cancers, and HR in men = 2.62 (1.90–3.62) and HR in women 1.49 (1.07–2.06) for alcohol-related cancers only. Heavy alcohol users (>5 drinks/day for men and >2.5 drinks/day for women) showed between 2 to 5 times higher risk of mortality of alcohol-related cancers, compared with light alcohol users (≤1 and ≤0.5 drink/week for men and women, respectively) [66]: HR 3.82 (2.09–6.97) in men and HR 2.20 (1.16–4.18) in women. Other beverages, such as soft drinks, increased the risk of mortality of colorectal cancer (HR 1.25, 1.07–1.47) [67] but not that of overall, breast, or prostate cancer [67], or renal cell carcinoma [68]. In addition, juice consumption increased renal cell carcinoma mortality in women: HR 1.17 (1.05–1.29) [68].

*Body fatness and height*: A high BMI (>35 kg/m^2^) compared to a low BMI (<23.5 kg/m^2^) was associated with an increased risk of all cancer mortality in women: HR 1.38 (1.14–1.68), but not in men [69]. Higher waist circumference (>103 cm in men and >89 cm in women) compared to lower waist circumference (<86 cm in men and <70 cm in women) was also associated with an increased risk of overall cancer mortality: HR in men = 1.89 (1.51–2.36) and HR in women = 1.30 (1.05–1.60) [69]. Annual weight loss (in an elderly population) [70] and height [71] were positively associated with risk of all cancer mortality: OR 4.57 (2.36–8.85), and HR in men = 1.11 (1.00–1.24) and HR in women = 1.17 (1.07–1.28). No associations were found between overall cancer mortality and any anthropometric measure of obesity among participants diagnosed with diabetes [72]. There were also no significant associations of television viewing time [73] and weight loss or weight gain [74] with cancer mortality.

*Other dietary exposures:* Eicosenoic and eicosapentaenoic acid intake increased the risk of prostate cancer mortality: HR 1.05 (1.00–1.11) and HR 1.07 (1.00–1.14), respectively; intake of other fatty acids was not associated with this outcome [75]. Finally, daily mean dietary greenhouse gas emissions were borderline associated with a higher risk of cancer mortality: HR 1.07 (0.99–1.15) [76].

**Table 3:** Dietary factors investigated as potential risk factors for cancer mortality in the EPIC study.

Tumour Site and EPIC Subcohort If Applicable	No. of Cases (No. of Deaths)	Mean Follow-Up (Years)	Results, Relative Risk [95% Confidence Interval (CI)]	Reference	
Cancer overall	448,568 (26,344 total deaths/9861 from cancer)	12.7 years since recruitment	No association between intake of red meat, processed meat, or poultry and cancer-related mortality risk. Very high consumption of red meat was associated with non-significantly increased cancer mortality (more than 160 g/d intake vs. low intake): HR 1.21 (1.00–1.46). Very high intakes of processed meat and poultry were also not significantly associated with this risk: HR 1.15 (0.90–1.46) and HR 1.00 (0.83–1.20), respectively.	Rohrmann 2013 [61]	
Cancer overall EPIC-Norfolk EPIC-Oxford	75,046 (8158 total deaths)	~17.0 years since recruitment	Tinned fruit consumption was not associated with cancer mortality (compared to the reference category of less than one serving of tinned fruit per month): HR 1.01 (0.90–1.12) and HR 1.07 (0.94–1.21) for one to three servings per month in EPIC-Norfolk and EPIC-Oxford, respectivey; HR 1.08 (0.94–1.24) and HR 0.87 (0.72–1.06) for one serving per week in EPIC-Norfolk and EPIC-Oxford, respectivey; and HR 0.90 (0.73–1.11) and HR 0.90 (0.70–1.17) for two or more servings per week in EPIC-Norfolk and EPIC-Oxford, respectivey.	Aasheim 2015 [60]	
Colorectal cancer	3789 (1262 total deaths/1008 from cancer)	4.1 years since diagnosis	No association between pre-diagnostic intakes of red meat and poultry and death due to colorectal cancer, for high vs. low intake: HR red and processed meat = 0.99 (0.84–1.15); HR red meat = 0.99 (0.89–1.10); HR processed meat = 1.00 (0.95–1.05); HR poultry = 0.96 (0.89–1.03). For processed meat and colorectal cancer mortality, a borderline significant trend was detected across quartiles (*p* = 0.053).	Ward 2016 [38]	
Cancer overall EPIC-Spain	41,199 (3316 total deaths)	18.0 years since recruitment	There was an association between Inflammatory Score of the Diet and cancer mortality (high vs. low score): HR 1.44 (1.22–1.69).	Agudo 2017 [63]	
Cancer overall Colorectal cancer Breast cancer Prostate cancer	451,743 (41,693 total deaths/18,003 from cancer)	16.4 years since recruitment	No association between intake of soft drinks and cancer mortality risk. By cancer site, total soft drink consumption was positively associated with colorectal cancer deaths (≥1 glass per day vs. <1 glass per month): HR 1.25 (1.07–1.47). No association between colorectal cancer mortality and sugar-sweetened or artificially sweetened soft drinks: HR 1.22 (0.91–1.64), and HR 1.10 (0.86–1.40), respectively. No associations were found for breast or prostate cancer mortality risk.	Mullee 2019 [67]	
Cancer overall EPIC-Spain	40,621 (3561 total deaths/1694 from cancer)	18.0 years since recruitment	No association was observed between egg consumption and cancer-related mortality (highest vs. lowest intake): HR 1.11 (0.96–1.28). HR per eggs/week = 1.01 (1.00–1.03).	Zamora-Ros 2019 [62]	
Cancer overall	501,594 (54,951 total deaths/23,143 from cancer)	17.2 years since recruitment	Those with a higher Food Standards Agency nutrient profiling system dietary index score (high vs. low score) showed an increased risk of mortality from cancer: HR 1.08 (1.03–1.13)	Deschasaux 2020 [64]	
Prostate cancer	7036 (936 deaths from cancer)	13.9 years since recruitment	No association between intake of individual fatty acids with death due to prostate cancer, except for eicosenoic and eicosapentaenoic acid (per 1 SD increase in intake): HR 1.05 (1.00–1.11) and HR 1.07 (1.00–1.14), respectively.	Perez-Cornago 2020 [75]	
Cancer overall EPIC-Spain	40,621 (3561 total deaths)	18.0 years since recruitment	A borderline association between daily mean dietary greenhouse emission (third vs. the first tertile) and cancer mortality risk was found: HR 1.07 (0.99–1.15).	González 2021 [76]	
Renal cell carcinoma	389,220 (356 deaths from cancer)	16.0 years since recruitment	Total and artificially sweetened soft drinks were positively associated with renal cell carcinoma mortality in models unadjusted for BMI and energy intake, but not after adjustment. Juice consumption was positively associated with renal cell carcinoma mortality in women, even after adjustment for BMI and energy intake (per 100 g/day increment): HR 1.17 (1.05–1.29)	Heath 2021 [68]	

HR: hazard ratio.

**Table 4:** Other lifestyle exposures investigated as potential risk factors for cancer mortality in the EPIC study.

Tumour Site and EPIC Subcohort If Applicable	No. of Cases (No. of Deaths)	Mean Follow-Up (Years)	Results, Relative Risk [95% Confidence Interval (CI)]	Reference
Cancer overall	359,387 (total deaths/5429 from cancer)	9.7 years since recruitment	Nonlinear association between BMI and cancer-related mortality risk. High BMI (>35 kg/m^2^) vs. low BMI (23.5 kg/m^2^): HR in men = 1.24 (0.97–1.60) and HR in women = 1.38 (1.14–1.68). Higher waist circumference was also associated with increased risk of cancer-related mortality. High (>103 cm in men and >89 cm in women) vs. low waist circumference (<86 cm in men and <70 cm in women): HR in men = 1.89 (1.51–2.36) and HR in women = 1.30 (1.05–1.60).	Pischon 2008 [69]
Cancer overall	34,239 (1712 total deaths)	7.0–15.0 years since recruitment	There was an association between annual weight loss by more than 1 kg and cancer death within 1 year in elderly population: OR 4.57 (2.36–8.85)	Bamia 2010 [70]
Cancer overall among diabetics (confirmed at recruitment)	5435 (641 total deaths/133 from cancer)	9.3 years since recruitment	No association between any anthropometric measure of obesity and cancer-related mortality risk in subjects diagnosed with diabetes. For instance, HR for obese (BMI > ~30kg/m2) vs. normal weight subjects (BMI< ~26 kg/m2) = 1.35 (0.70–2.58) in men and HR 1.46 (0.63–3.40) in women	Sluik 2011 [72]
Cancer overall EPIC-Norfolk	13,197 (1270 total deaths/570 from cancer)	9.5 years since recruitment	No association between television viewing time and cancer-related mortality risk (per 1 h/day increase in television time): HR 1.04 (0.98–1.10).	Wijndaele 2011 [73]
Cancer overall among diabetics (confirmed at recruitment)	4797 (533 total deaths/109 from cancer)	9.2 years since recruitment	No association between alcohol intake in the past and cancer-related mortality risk in subjects diagnosed with diabetes. For instance, HR for high (6 g/d intake) vs. low alcohol intake (abstainers: 0 g/d intake) = 0.88 (0.47–1.65) in men and HR 0.54 (0.19–1.54) in women.	Sluik 2012 [77]
Alcohol-related cancers	380,395 (26,411 total deaths/2764 from cancer)	12.6 years since recruitment	Heavy alcohol users (>5 drinks/day for men and >2.5 drinks/day for women), regardless of time of cessation, had a 2 to 5 times higher risk of dying due to alcohol-related cancers, compared with subjects with lifetime light alcohol use (≤1 and ≤0.5 drink/week for men and women, respectively): HR 3.82 (2.09–6.97) in men and HR 2.20 (1.16–4.18) in women.	Bergmann 2013 [66]
Cancer overall Alcohol-related cancers	380,395 (20,453 total deaths/2053 from cancer)	12.6 years since recruitment	In men, extreme alcohol use (≥60 g/day) compared to moderate drinkers (0.1–4.9 g/day) was associated with mortality due to alcohol-related cancers and with other cancers: HR 2.62 (1.90–3.62) and HR 1.34 (1.13–1.59), respectively. Among women, heavy drinkers (≥30 g/day) compared to moderate drinkers (0.1–4.9 g/day) were associated with mortality due to alcohol-related cancers: HR 1.49 (1.07–2.06).	Ferrari 2014 [65]
Cancer overall	409,748 (29,810 total deaths/11,931 from cancer)	12.5 years since recruitment	Height was positively associated with cancer mortality (highest vs. lowest quartile): HR in men = 1.11 (1.00–1.24) and HR in women = 1.17 (1.07–1.28). Sitting height was not associated with cancer mortality.	Sawada 2017 [71]
Cancer overall EPIC-Norfolk	12,580 (2603 total deaths/981 from cancer)	15.0 years since recruitment	Neither weight loss nor weight gain were associated with risk of cancer-related mortality in men (for >5 kg): HR 1.45 (0.98–2.15) and HR 1.34 (0.66–2.72), respectively. In women, associations were also not significant (for >5 kg): HR for weight loss = 1.36 (0.92–2.01) and HR for weight gain = 0.64 (0.26–1.55).	Mulligan 2018 [74]

BMI: body mass index; HR: hazard ratio; OR: odds ratio.

## 4. Discussion

This is the first review summarizing findings from the EPIC study on the association between dietary factors and other lifestyle exposures with overall cancer mortality and cause-specific mortality. Although many potentially protective diet-related exposures were examined in EPIC, only a few demonstrated significant effects on cancer mortality, including raw vegetable intake, dietary fiber intake, and intake or plasma levels of some vitamins, such as vitamin D. In general, associations with individual foods were rarely significant, whereas dietary patterns accounting for intake of several foods were significantly associated with lower cancer mortality. For instance, higher adherence to the Mediterranean diet and to other diet quality indexes were found to reduce overall cancer mortality risk. In contrast, alcohol intake and obesity were consistently associated with an increased risk of mortality due to cancer. Nevertheless, there were also some ambiguos results, with some studies finding significant associations and others, null results. 

### 4.1. Protective Factors against Cancer Mortality

The lack of associations between intake of fruits, vegetables, and legumes and cancer mortality observed in EPIC [33,34] is in agreement with the results reported in a broader systematic review evaluating this association with regard to fruits and vegetables (*n* = 16,817 cancer deaths) [78] and with those reported with regard to legumes in the PREDIMED study (*n* = 169 cancer deaths) [79]. In contrast, in another review, higher intake of vegetables was inversiley associated with overall mortality, though among cancer survivors only, at both cancer pre- and postdiagnosis [19]. Importantly, when considering cancer-specific mortality, it has been shown that intake of vegetables could reduce cancer mortaltiy in survivors of head and neck cancer and of ovarian cancer, but not other cancer types [80]. In the EPIC study, only a few cancer-specific associations were evaluated and, for instance, there was a non-significant association between intake of fruits and vegetables and mortality related to prostate cancer [35].

A relevant research result from the EPIC study is that intake of raw vegetables seems to decrease cancer mortality. It has been suggested that raw versus cooked vegetables could have a stronger effect on cancer risk due to their higher nutrient availability [81], but no other studies have evaluated this association in relation to cancer mortality. Associations of other plant-based foods with cancer mortality have not yet been examined in EPIC, with the exception of olive oil [58]. Concerning bioactive compounds contained in these foods, a number of significant associations with cancer mortality have been reported in the EPIC study, namely, for fibre intake [36,37] and for lignan intake, the latter being associated with reduced cancer mortality in breast cancer survivors [56]. A high intake of dietary fibre was mostly associated with lower mortality of smoking-related cancers [37]. It is also important to highlight that dietary fibre intake was not associated with mortality among those diagnosed with colorectal cancer [38], despite current evidence supporting that high-fibre foods are likely to reduce colorectal cancer risk [5]. Moreover, no significant associations were found for intake of total flavonoids and lignans and overall cancer mortality risk [59]. A meta-analysis that combined these results together with those of three further non-EPIC studies reaffirmed this lack of association between both intake of flavonoids and cancer mortality [82]. 

Other bioactive compounds, such as antioxidants, could also exert protective effects againts cancer mortality by mitigating the impact of oxidative stress on the body. A potential association between non-recent use of antioxidant vitamin supplements and vitamin C and cancer mortality prevention was observed in EPIC [55], but this association remains controversial in light of a research study conducted in the UK-Biobank cohort (*n* = 10,780 cancer deaths) that found different results [83]. A diet rich in vitamin K was also associated with reduced mortality for all cancers in one EPIC study [52], but not in another country-based EPIC study [53]. 

Intake of foods from animal origin, including fish, milk, and dairy products were not associated with cancer mortality in the EPIC study [39,40], in agreement with the results of a systematic review on this issue [19]. Only in the study by Langlais et al. was consumption of whole milk/high-fat dairy associated with higher risk of prostate cancer recurrence and mortality [84]. Thus, whether consumption of these foods is associated with cancer-specific mortality remains to be explored further. Dairy products are a source of dietary calcium and vitamin D, both of which have been examined in relation to cancer mortality, too. One of the primary results reported by EPIC showed that high plasma levels of vitamin D at cancer pre-diagnosis could reduce mortality of colorectal and renal cancer [50,51]. Other studies also support that vitamin D lowers mortality of various cancer types, including colorectal cancer [85]. The mechanisms by which vitamin D could increase cancer survival go through the activation of different molecular pathways to inhibit tumour cell proliferation, growth, invasiveness, and inflammatory signalling, among others [86]. Similarly, these mechanims could underly the association between vitamin D and colorectal cancer risk, an inverse association that has been shown in some studies [5], including the EPIC study [87].

Higher Mediterranean diet scores were related to reduced overall cancer mortality in the EPIC study [42], and this association was found to be stronger for cancers of the digestive tract [41]. The Mediterranean diet is a plant-based food dietary pattern characterized by high intake of fruits and vegetables, nuts and seeds, cereals and legumes, frequent consumption of fish and seafood, and moderate intake of wine [88]. This dietary pattern, considered healthy for its high fiber and antioxidant count, might have a beneficial effect in reducing cancer mortality, as reflected in several studies [89]. Likewise, an overall plant-based food dietary pattern, and specifically either vegetarian or vegan diet, was inversely associated with cancer mortality in the EPIC study [43]. This association was stronger for death due to pancreatic and lymphatic/hematopoietic cancers [43,44]. While there are some conflicting results in the literature regarding the association of these diets with cancer mortality, there is a general trend towards a protective association [89]. Concerning other diet quality indexes also involving higher consumption of vegetables, fruits, legumes, nuts, whole grains, vegetable oils, fish, and lean meat or poultry, many studies support an inverse association between increasing adherence to diet quality scores and lower cancer mortality [90]. 

In the EPIC study, healthy dietary patterns in combination with other lifestyle factors, expressed in combined adherence/lifesyle scores, were associated with reduced overall cancer mortaliy [42,48,49]. This association was maintained after pooling the results of EPIC and two other studies [91]. By cancer type, in the latter studies, adherence to the WCRF recommendations was associated with lower breast, colorectal, and lung cancer mortality [91,92], though not with mortality from other cancers [91,93]. The EPIC study also encountered an association between high adherence to the WCRF score and lower colorectal cancer mortality; however, associations with other cancer types were not examined. Overall, as in the EPIC study, these studies assessed how pre-diagnosis diet and lifetyle recommendations affect cancer mortality. The WCRF recommendations include physical activity as a major lifestyle component, due to its well-known anticancer effects (lower BMI and adiposity; lower sex hormones, insulin, and c-peptide levels; and decreased inflammation and immune response) [5,94]. Indeed, with respect to cancer mortality, the EPIC study also demonstrated that physical exercise, in general and before cancer diagnosis, prevents cancer mortaltiy [45].

### 4.2. Risk Factors for Cancer Mortality

Foods of animal origin have not been clearly linked to cancer mortality in the EPIC study. The current evidence, as summarized in the WCRF/AICR report, supports intake of red and processed meat as contributing to colorectal cancer development [5]. However, findings from the EPIC study do not show that these effects extend to colorectal cancer mortality [38,39]. These foods are rich in saturated fatty acids, heme iron, nitrites, and nitrosamines, all of which might activate the carcinogenic process through several biological mechanisms [95]. 

Intake of saturated fatty acids was not associated with prostate cancer mortality in the EPIC study, but butiric acid was associated with advanced-stage disease [75]. On the other hand, mortality risk of this cancer tended to increase modestly with increasing intakes of eicosenoic (22:1n--9c) and eicosapentaenoic acid (20:5n--3c) [75]. Both are polyunsatured fatty acids, mainly found in fish, nuts, and seeds. An increased risk of fatal prostate cancer was observed for high intakes of saturated fats within the NIH-AARP study, but not so for eicosapentanoit acid (*n* = 725 prostate cancer deaths) [96]. So far, these associations have not been evaluated for other cancer types in the EPIC study. Similarly, cancer mortaltiy associations with other components of red and processed meat have not yet been evaluated. Other foods of animal origin, such as eggs, were not associated with cancer mortality in EPIC [62].

Poor diet quality as measured by the diet inflammation index was associated with overall cancer mortality [63]. In general, diets high in carbohydrates and saturated fats and low in fiber seem to have proinflammatory effects that promote cell proliferation, DNA damage, and immune activation [97], thereby leading to worse cancer outcomes. Within the US National Health and Nutrition Examination Survey (NHANES), a positive association was observed between high vs. low diet inflammation index and overall cancer mortality (*n* = 490 cancer deaths) [98], further supported by a meta-analysis of six prospective studies on this issue [97]. This association was also strong and consistent for overall cancer mortality in the EPIC study [63], although cancer-specific mortality outcomes have not been examined. 

Alcohol intake and obesity are well-established risk factors for cancer development acccording to the WCRF/AICR Third Export Report [5]. In relation to overall cancer mortality, the EPIC study found positive associations for alcohol intake [65,66], BMI, abdominal obesity [69], and weight loss in the elderly [70], in both sexes. In patients diagnosed with diabetes, however, there was no association between obesity and overall cancer mortality, possibly due to the metabolic consequences of diabetes [72]. Obesity is associated with dysregulation of multiple metabolic risk factors such as insulin resistance and a low-grade chronic inflammation state, which are also associated with increased cancer risk and poor prognosis [99]. 

Concerning alcohol consumption, heavy alcohol drinkers were found to be at a higher risk for mortality from alcohol-related cancers [66]. Many studies have been undertaken to characterize the association between alcohol consumption and cancer mortality. Together, these studies support that alcohol consumption has detrimental effects on overall and cancer-specific mortality [100,101]. Alcoholic beverages contain acetaldehyde and ethanol, both of which seem to have genotoxic and inflammation effects that drive the carcinogenic process [102]. For other sugary beverages, the EPIC study reported positive associations between consumption of sweetened beverages and colorectal cancer mortality only and between consumption of juices and renal cancer, whereas no association was observed for overall cancer mortality or mortality from other cancer types [67]. This finding is supported by a recent meta-analysis that showed non-significant associations between sugar-sweetened and artificially sweetened beverages or juices with overall cancer mortality [103]. Finally, null results have been reported by the EPIC study with regard to other dietary factors, such as tinned fruit [60] and daily mean dietary greenhouse emission [76].

As stated before, this is the first review to summarize results of all EPIC studies to date on dietary factors in relation to cancer mortality. There are, nevertheless, several limitations that should be noted. First, a rapid review was conducted, which means that some steps of the standard Systematic Review approach can be avoided. This kind of review is, therefore, subject to bias. However, we conducted an assessment of the methodological quality of all included studies, similar to that performed in our previous systematic review on cancer incidence in the EPIC study [24]. Similarly, this assessment resulted in generally high and highly homogenous scores. All studies assessed associations between exposures at recruitment and cancer mortality during follow-up. Hence, none of the studies accounted for the influence of prognostic factors, such as cancer treatment and stage of the tumour at diagnosis. In addition, exposure changes after cancer diagnosis and associations between post-diagnosis dietary-related factors and cancer mortality were not evaluated in any study. A small number of studies evaluated the associations between exposures and mortality risk starting follow-up from the date of diagnosis of the tumour, i.e., only cancer survivors were studied [38,49,50,51,56]; however, these studies, too, considered the pre-diagnostic exposures measured at recruitment.

Finally, cancer recurrence or development of second cancers were not investigated in any study and only a few studies evaluated mortality by cancer site, with prostate cancer and colorectal cancer mortality being the most frequent outcomes. The limited statistical power to detect significant associations in cancer-specific mortality studies of less common cancers is another important limiting factor that makes it difficult to draw meaningful conclusions about the association between diet and lifestyle factors with cause-specific mortality. 

## 5. Conclusions

The EPIC study has unravelled several dietary factors and other lifestyle exposures that influence cancer mortality and that, if confirmed in other studies, will serve to strengthen cancer prevention recommendations. Findings from EPIC support that adherence to healthy dietary patterns, mantaining normal weight, and engaging in regular physical activity can prevent cancer mortality and support current cancer prevention recommendations set out by the WCRF/AICR. However, more specific evidence-based recommendations for cancer survivors are needed. Thus, while these findings are important, it is equally important to take account of the fact that much remains to be done to elucidate the role of diet, obesity, and physical activity in cancer mortality prevention.

## Data Availability

Not applicable.

## Supplementary Materials

The following are available online at https://www.mdpi.com/article/10.3390/nu13124293/s1. Table S1: Search terms used in databases; Table S2: Joanna Briggs Institute Critical Appraisal Tool for Cohort Studies; and Table S3: Quality assessment of included articles.
