# Supplementary material for: The Role of Diet, Alcohol, BMI, and Physical Activity in Cancer Mortality: Summary Findings of the EPIC Study
Source: Nutrients. 2021 Nov 28;13(12):4293. doi: 10.3390/nu13124293 (PMC8709081; doi:10.3390/nu13124293)
Supplement: Supplementary file 1 [file nutrients-13-04293-s001.zip › nutrients-1465637-supplementary.pdf]

SUPPLEMENTARY MATERIAL

Table S1. Search terms used in databases.

**MEDLINE (via PubMed)**
(((("european prospective investigation into cancer" OR "EPIC study") AND ("cancer" OR "tumor" OR "tumour" OR "myelo*" OR "leukaemia" OR "leukemia" OR "neoplasm*" OR "lympho*" OR "carcinoma" OR "sarcoma") AND ("diet" OR "intake" OR "nutrients" OR "physical activity" OR "exercise" OR “BMI” OR “alcohol”) AND (“mortality” OR “survival”))))
**Scopus**
TITLE-ABS-KEY (("european prospective investigation into cancer" OR "EPIC study") AND ("cancer" OR "tumor" OR "tumour" OR "myelo*" OR "leukaemia" OR "leukemia" OR "neoplasm*" OR "lympho*" OR "carcinoma" OR "sarcoma") AND ("diet" OR "intake" OR "nutrients" OR "physical activity" OR "exercise" OR "BMI" OR "alcohol") AND ("mortality" OR "survival"))
**Web of Science**
(("european prospective investigation into cancer" OR "EPIC study")) AND TEMA: (("cancer" OR "tumor" OR "tumour" OR "myelo*" OR "leukaemia" OR "leukemia" OR "neoplasm*" OR "lympho*" OR "carcinoma" OR "sarcoma")) AND TEMA: (("diet" OR "intake" OR "nutrients" OR "physical activity" OR "exercise" OR “BMI” OR “alcohol”)) AND TEMA: (("mortality” OR “survival”))

Table S2. Joanna Briggs Institute Critical Appraisal Tool for Cohort Studies.

	Yes	No	Unclear	Not applicable
1. Were the two groups similar and recruited from the same population?	☐	☐	☐	☐
2. Were the exposures measured similarly to assign people to both exposed and unexposed groups?	☐	☐	☐	☐
3. Was the exposure measured in a valid and reliable way?	☐	☐	☐	☐
4. Were confounding factors identified?	☐	☐	☐	☐
5. Were strategies to deal with confounding factors stated?	☐	☐	☐	☐
6. Were the groups/participants free of the outcome at the start of the study (or at the moment of exposure)?	☐	☐	☐	☐
7. Were the outcomes measured in a valid and reliable way?	☐	☐	☐	☐
8. Was the follow up time reported and sufficient to be long enough for outcomes to occur?	☐	☐	☐	☐
9. Was follow up complete, and if not, were the reasons to loss to follow up described and explored?	☐	☐	☐	☐
10. Were strategies to address incomplete follow up utilized?	☐	☐	☐	☐
11. Was appropriate statistical analysis used?	☐	☐	☐	☐

Table S3. Quality assessment of included articles.

Study	Item 1	Item 2	Item 3	Item 4	Item 5	Item 6	Item 7	Item 8	Item 9	Item 10	Item 11	Quality Category
Aasheim 2015 [60]	N/A	N/A	✓	✓	✓	✓	✓	✓	✓	N/A	✓	High
Agudo 2017 [63]	N/A	N/A	✓	✓	✓	✓	✓	✓	✓	N/A	✓	High
Appleby 2016 [43]	N/A	N/A	✓	✓	✓	✓	✓	✓	✓	N/A	✓	High
Bamia 2010 [70]	✓	✓	✓	✓	✓	✓	✓	✓	✓	N/A	✓	High
Bergmann 2013 [66]	N/A	N/A	✓	✓	✓	✓	✓	✓	✓	N/A	✓	High
Buckland 2011 [41]	N/A	N/A	✓	✓	✓	✓	✓	✓	✓	N/A	✓	High
Buckland 2012 [58]	N/A	N/A	✓	✓	✓	✓	✓	✓	✓	N/A	✓	High
Burger 2012 [36]	N/A	N/A	✓	✓	✓	✓	✓	✓	✓	N/A	✓	High
Chuang 2012 [37]	N/A	N/A	✓	✓	✓	✓	✓	✓	✓	N/A	✓	High
Deschasaux 2020 [64]	N/A	N/A	✓	✓	✓	✓	✓	✓	✓	N/A	✓	High
Engeset 2015 [39]	N/A	N/A	✓	✓	✓	✓	✓	✓	✓	N/A	✓	High
Fedirko 2012 [50]	N/A	N/A	✓	✓	✓	✓	✓	✓	✓	N/A	✓	High
Ferrari 2014 [65]	N/A	N/A	✓	✓	✓	✓	✓	✓	✓	N/A	✓	High
González 2021 [76]	N/A	N/A	✓	✓	✓	✓	✓	✓	✓	N/A	✓	High
Heath 2021 [68]	N/A	N/A	✓	✓	✓	✓	✓	✓	✓	N/A	✓	High
Huerta 2016 [47]	N/A	N/A	✓	✓	✓	✓	✓	✓	✓	N/A	✓	High
Kyrø 2015 [56]	N/A	N/A	✓	✓	✓	✓	✓	✓	✓	N/A	✓	High
Lassale 2016 [42]	N/A	N/A	✓	✓	✓	✓	✓	✓	✓	N/A	✓	High
Li 2011 [57]	N/A	N/A	✓	✓	✓	✓	✓	✓	✓	N/A	✓	High
Li 2012 [55]	N/A	N/A	✓	✓	✓	✓	✓	✓	✓	N/A	✓	High
Leenders 2013 [34]	N/A	N/A	✓	✓	✓	✓	✓	✓	✓	N/A	✓	High
Mok 2019 [45]	N/A	N/A	✓	✓	✓	✓	✓	✓	✓	N/A	✓	High
Mullee 2019 [67]	N/A	N/A	✓	✓	✓	✓	✓	✓	✓	N/A	✓	High
Muller 2014 [51]	✓	✓	✓	✓	✓	✓	✓	✓	✓	N/A	✓	High
Mulligan 2018 [74]	N/A	N/A	✓	✓	✓	✓	✓	✓	✓	N/A	✓	High
Myint 2019 [54]	N/A	N/A	✓	✓	✓	✓	✓	✓	✓	N/A	✓	High
Nimptsch 2010 [52]	N/A	N/A	✓	✓	✓	✓	✓	✓	✓	N/A	✓	High
Nöthlings 2008 [33]	N/A	N/A	✓	✓	✓	✓	✓	✓	✓	N/A	✓	High
Pala 2019 [40]	N/A	N/A	✓	✓	✓	✓	✓	✓	✓	N/A	✓	High
Perez-Cornago 2017 [35]	N/A	N/A	✓	✓	✓	✓	✓	✓	✓	N/A	✓	High
Perez-Cornago 2020 [75]	N/A	N/A	✓	✓	✓	✓	✓	✓	✓	N/A	✓	High
Pischon 2008 [69]	N/A	N/A	✓	✓	✓	✓	✓	✓	✓	N/A	✓	High
Rohrmann 2013 [61]	N/A	N/A	✓	✓	✓	✓	✓	✓	✓	N/A	✓	High

Romaguera 2015 [49]	N/A	N/A	✓	✓	✓	✓	✓	✓	✓	N/A	✓	High
Sahlqvist 2013 [46]	N/A	N/A	✓	✓	✓	✓	✓	✓	✓	N/A	✓	High
Sawada 2017 [71]	N/A	N/A	✓	✓	✓	✓	✓	✓	✓	N/A	✓	High
Segovia-Siapco 2018 [44]	N/A	N/A	✓	✓	✓	✓	✓	✓	✓	N/A	✓	High
Sluik 2011 [72]	N/A	N/A	✓	✓	✓	✓	✓	✓	✓	N/A	✓	High
Sluik 2012 [77]	N/A	N/A	✓	✓	✓	✓	✓	✓	✓	N/A	✓	High
Vergnaud 2013 [48]	N/A	N/A	✓	✓	✓	✓	✓	✓	✓	N/A	✓	High
Ward 2016 [38]	N/A	N/A	✓	✓	✓	✓	✓	✓	✓	N/A	✓	High
Wijndaele 2011 [73]	N/A	N/A	✓	✓	✓	✓	✓	✓	✓	N/A	✓	High
Zamora-Ros 2013 [59]	N/A	N/A	✓	✓	✓	✓	✓	✓	✓	N/A	✓	High
Zamora-Ros 2019 [62]	N/A	N/A	✓	✓	✓	✓	✓	✓	✓	N/A	✓	High
Zwakenberg 2017 [53]	N/A	N/A	✓	✓	✓	✓	✓	✓	✓	N/A	✓	High
**Criterion Score %**	100	100	100	100	100	100	100	100	100	N/A	100	

Note that the criterion score is calculated by dividing the number of studies meeting one criterion by the total number of studies . ✓: meet the methodological quality criterion; ×: not meet the methodological quality criterion.; ?: uncelar; N/A: not applicable.
